# Cognitive reserve: Investigate the efficacy of resistance training on cognitive reserve in older adults with subcortical ischemic vascular cognitive impairment

**DOI:** 10.1002/alz.093314

**Published:** 2025-01-09

**Authors:** Yi Gu, Chun Liang Hsu, Narlon Cassio Boa Sorte Silva, Roger Tam, Walid Ahmed Alkeridy, Kevin Lam, Teresa Liu‐Ambrose

**Affiliations:** ^1^ Centre for Aging SMART, Vancouver Coastal Health Research Institute, Vancouver, BC Canada; ^2^ University of British Columbia, Vancouver, BC Canada; ^3^ Djavad Mowafaghian Centre for Brain Health, Vancouver, BC Canada; ^4^ The Hong Kong Polytechnic University, Hong Kong Hong Kong; ^5^ King Saud University, Riaydh Saudi Arabia

## Abstract

**Background:**

Subcortical ischemic vascular cognitive impairment (SIVCI) is the most common form of vascular cognitive impairment. White matter hyperintensities (WMH) secondary to SIVCI often result in impaired executive function. Cognitive reserve is a property of the brain that allows for preserved cognitive performance given brain injury or disease. In an unpublished cross‐sectional study, we found the strength of intra‐network connectivity in fronto‐executive network (FEN), fronto‐parietal network (FPN) and default mode network (DMN) moderated the relationship between WMH and executive function in older adults with SIVCI. Thus, these networks may be involved in cognitive reserve. Exercise is hypothesized to contribute to cognitive reserve. We explored the effect of resistance training (RT) on functional connectivity (FC) in FEN, FPN, and DMN, as well as executive function, in older adults with SIVCI.

**Method:**

In a 12‐month randomized controlled trial that randomized adults with SIVCI to either 2x/week of RT or 2x/week of balance and tone training (BAT; control). Resting‐state functional MRI was collected from a subset of 29 participants (RT = 14; BAT = 15) at baseline and trial completion. FC of FEN, FPN, and DMN was quantified using priori‐selected region‐of‐interest masks. Executive function was measured by Trail Making Test (TMT), Digit Symbol Substitution Test (DSST) and Stroop Test (ST). Analysis of covariance was conducted to compare differences in group means of intra‐network FC of FEN, FPN and DMN and executive function tests performance at trial completion, with baseline outcome scores as covariate. Pearson correlations were computed to determine whether changes in executive performance between baseline and trial completion were related to changes in FC in the RT group.

**Result:**

At trial completion, there were no significant between‐group differences in intra‐network FC of FEN, FPN and DMN. RT significantly improved ST performance (estimated mean change[95%CI]: ‐11.322[‐22.366, ‐0.277], *p* = 0.045) compared with BAT; there were no significant between‐group differences in TMT and DSST. Within RT group, improved ST performance was significantly correlated with increased intra‐network FC of FEN (*r* = ‐0.697, *p* = 0.006).

**Conclusion:**

RT‐induced improvement in ST performance was associated with increased intra‐network FC of FEN. More research is needed to determine whether exercise training has an effect on the neural correlates of cognitive reserve.

